# Disease-modifying therapies and hematological disorders: a systematic review of case reports and case series

**DOI:** 10.3389/fneur.2024.1386527

**Published:** 2024-06-18

**Authors:** Cristina Scavone, Valerio Liguori, Olusola Jephthah Adungba, Daniele Di Giulio Cesare, Maria Giuseppa Sullo, Vincenzo Andreone, Liberata Sportiello, Giorgia Teresa Maniscalco, Annalisa Capuano

**Affiliations:** ^1^Department of Experimental Medicine, University of Campania “Luigi Vanvitelli”, Naples, Italy; ^2^Regional Center of Pharmacovigilance and Pharmacoepidemiology of Campania Region, Naples, Italy; ^3^Eu2P Programme, University Bordeaux, Bordeaux, France; ^4^Multiple Sclerosis Regional Center, “A. Cardarelli” Hospital, Naples, Italy; ^5^Neurological Clinic and Stroke Unit, “A. Cardarelli” Hospital, Naples, Italy

**Keywords:** multiple scleorsis, DMT, hematological disorders, systematic reveiw, case reports, case series

## Abstract

**Introduction:**

Disease modifying therapies (DMTs) used to treat multiple sclerosis (MS) can be associated to the occurrence of hematological disorders. This systematic review aims to provide an overview of these events occurring in real-life conditions, by describing case reports and series published in the literature.

**Methods:**

A literature search of all publications up to January 5th 2024 on the Medline and Embase databases was carried out. The results were presented both in the text and in tables.

**Results:**

Sixty-seven case reports/series were included in this review, of which more than half related to alemtuzumab, natalizumab and ocrelizumab. The publication date of included studies ranged from 2006 to 2024. The majority of case reports and series described the occurrence of late-onset hematological disorders (events that occurred more than 30 days after the first DMT administration), mainly represented by case of neutropenia, autoimmune hemolytic anemia and immune thrombocytopenia. All cases reported a favorable outcome, apart one case report that described a fatal case. Among included cases, 4 articles, all related to natalizumab, described the occurrence of myeloid disorders in 13 newborns from mother receiving the DMT.

**Discussion:**

Considering the limitations identified in the majority of included studies, further *ad hoc* studies are strongly needed to better evaluate the hematological disorders of DMTs. Meantime, the strict monitoring of treated patients for the occurrence of these toxicities should be highly recommended.

## 1 Introduction

Multiple sclerosis (MS) is a chronic, autoimmune disease affecting the central nervous system (CNS) which is the result of an immune dysregulation associated with genetic and environmental factors (1). Clinically isolated syndrome (CIS) refers to a first episode of neurologic symptoms (that might include optic neuritis, vertigo, weakness in the arms and legs, difficulty with coordination, balance, walking, speaking and ataxia) caused by inflammation in the CNS that could become MS if additional activity occurs (2). According to the 2017 revision of McDonald criteria (3), the diagnosis of MS can be made in patients with CIS and clinical or MRI demonstration of dissemination in space, and in presence of Cerebrospinal Fluid (CSF)-specific oligoclonal bands. Once MS is diagnosed, different subtypes can be distinguished, including relapsing remitting MS (RRMS), primary progressive MS (PPMS) and secondary progressive MS (SPMS). Among these forms, RRMS is the most common subtype (4). Based on this classification, RRMS is characterized by periods between relapses that are free of worsening, while MS progressive forms present a period during which patients exhibit continuous decline of neurological functions. However, as recently reported by Granziera et al. (5) steady progression independent of relapse activity (PIRA) is a frequently identified in RRMS. Recent data from an open-source global compendium on MS epidemiology reported that 2.8 million people are estimated to live with MS worldwide (35.9 per 100,000 population) and that since 2013 MS prevalence has increased globally (including among pediatric population). The pooled incidence rate is 2.1 per 100,000 persons/year (6). The mean age at diagnosis is 32 years, and the combined incidence rate among the 75 reporting nations is 2.1 per 100,000 people/year. The disease is twice as common in women as it is in men, although the ratio of women to men is as high as 4:1 in some countries (6, 7).

The pharmacological management of MS foresees the use of disease-modifying therapies (DMTs) that are able to reduce the number of relapses and delay disease’s progression (8). These drugs, which act on different biological pathways and show distinct efficacy/safety profiles, are classified as low/moderate- or high-efficacy treatment. Among low/moderate efficacy therapies are IFNBs, glatiramer acetate (GA), teriflunomide, and dimethyl fumarate (DMF). These therapies are generally safer than higher efficacy agents. On the other hand, monoclonal antibodies (ocrelizumab, natalizumab, alemtuzumab and ofatumumab), Sphingosine 1-phosphate receptor (S1PR) modulators (fingolimod, siponimod, ozanimod, and ponesimod), cladribine and mitoxantrone show higher efficacy profiles but they are also associated with greater risks of adverse drug reactions (ADRs) (9, 10). DMTs have distinct pharmacodynamics properties, resulting in immunomodulatory and anti-inflammatory response, and consequently impact individual efficacy and tolerability profiles. A recent study carried out by Barbieri MA et al. on data from the Italian Pharmacovigilance database highlighted that the most reported DMTs-induced ADRs were general and administration site conditions, followed by nervous, skin and blood disorders (11). Among blood disorders, myelosuppression, also defined as myelotoxicity or bone marrow suppression, represents a rare idiosyncratic ADR that can be associated with any drug, including DMTs. Myelotoxicities, which include anemia, leucopenia and thrombocytopenia, are potentially life-threatening events due to infection and bleeding complications of neutropenia and thrombocytopenia (12). As reported by Schweitzer et al., DMTs are able to selectively suppress or modulate the immune system leading to unwanted ADRs affecting leukocytes in peripheral blood (13).

In order to provide an overview of DMTs-induced hematological disorders in real-life conditions, we carried out an extensive systematic review of published case reports and case series. Our aims are to describe the main characteristics of DMTs-induced hematological disorders in terms of patients’ demographic, their medication history, suspected DMTs, seriousness, management and outcome of the event and time of onset of selected ADRs and to provide general evidence regarding the hematological profile of DMTs currently used to treat MS.

## 2 Materials and methods

### 2.1 Search strategy

The Preferred Reporting Items for Systematic Reviews and Meta-Analysis (PRISMA) guideline (14) and the Cochrane Handbook for Systematic Reviews of Interventions (v6.4) (15) were used to perform a standardized data search, extraction, reporting, and presentation.

Two authors (CS and VL) independently performed a literature search of all publications up to January 5th, 2024 on the Medline and Embase databases using the following keywords: multiple sclerosis AND (aplastic anemia OR anemia OR neutropenia OR thrombocytopenia OR myelosuppression OR pancytopenia) AND (disease-modifying therapies OR DMT OR DMTs OR alemtuzumab OR interferon beta-1b OR cladribine OR teriflunomide OR glatiramer acetate OR ofatumumab OR peginterferon beta-1a OR fingolimod OR siponimod OR ozanimod OR natalizumab OR ponesimod OR ocrelizumab OR dimethyl fumarate OR diroximel fumarate OR interferon beta-1a) AND (case report OR case series OR clinical case OR clinical cases).

### 2.2 PICOS/study selection

Population: patients diagnosed with MS according to Mc Donald diagnostic criteria (3); Interventions: treatment with a DMT approved for the MS (thus, excluding DMTs used in off-label conditions, such as rituximab); Comparators: none; Outcomes: occurrence of myelo-lymphoid ADRs such as aplastic anemia, anemia, neutropenia, thrombocytopenia, myelosuppression, pancytopenia (including hematologic autoimmune conditions such as Autoimmune hemolytic anemia - AIHA - and immune thrombocytopenia - ITP) in patients receiving a DMT; Study designs: case reports or case series.

Screening was based on reading the titles, abstracts and full-texts of the publications. Articles not in English language were excluded. We also excluded meta-analyses, reviews, meeting/conference abstracts, clinical trials and observational studies.

### 2.3 Data extraction and analysis

Data on selected articles were imported into MS Excel. Data were collected from the full-text publications relevant to DMTs-induced myeloid ADRs, including patient demographics, medication history for MS, time to event [(TTE), the intervening period of time from the first DMT administration to the occurrence of myeloid ADR], ADRs’ signs and symptoms, including relevant laboratory findings related to myeloid toxicity, seriousness and outcome.

### 2.4 Risk of bias assessment of individual studies

A quality assessment of the included studies was performed using the Joanna Briggs Institute (JBI) critical appraisal checklist for case reports and series (16). For neonatal cases the risk of bias assessment was not performed. Any discrepancies were resolved through discussion.

## 3 Results

**Figure 1:**
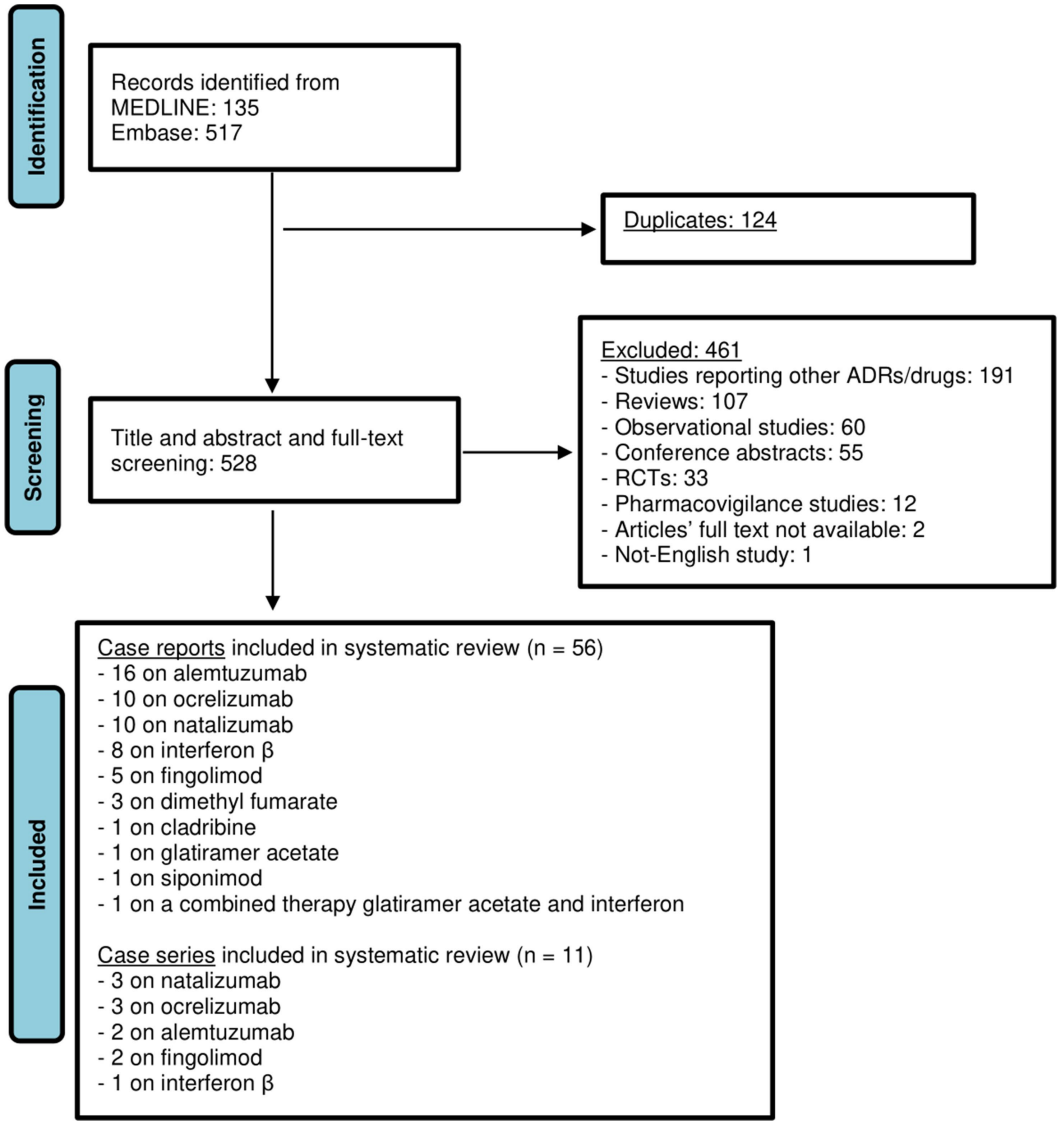
Flow chart illustrating literature search outcomes. From: Page MJ, McKenzia JE, Bossuyt PM, Boutron I, Hoffmann TC, Mulrow CD, et al. The PRISMA 2020 statement: an updated guideline for reporting systematic reviews. BMJ 2021:372:n71. doi: 10.1136/bmj.n71. For more information, visit: http://www.prisma-statement.org/.

Our initial search yielded 135 results from Medline and 517 results from Embase. Following the screening of title, abstract and a more thorough examination of full text, 67 articles were included in this review (17–83), of which 56 case reports and 11 case series concerning overall 97 patients (84 adults and 13 newborns). Of included studies, 18 were related to alemtuzumab (*n* = 20 patients), 13 to natalizumab (*n* = 11 adults; *n* = 13 newborns), 13 to ocrelizumab (*n* = 25), 9 to IFN (*n* = 10), 7 to fingolimod (*n* = 11), 3 to DMF (*n* = 3), 1 to cladribine (*n* = 1), 1 to siponimod (*n* = 1), 1 to glatiramer acetate (*n* = 1) and 1 to the combined therapy GA and IFN-β 1a (*n* = 1) (Figure 1; Tables 1–3). Among these cases, 4 articles (29, 49, 50, 55), all related to natalizumab, described the occurrence of myelotoxicities in newborns from mother receiving the DMT. In the Supplementary Table S1 an overview of DMTs, including their routes and frequencies of administration, mechanisms of actions and data from clinical trials on the occurrence of hematological disorders are reported.

**Table 1:** Overview of DMTs-induced early-onset myelotoxicities.

DMT (ref)	Age (years), sex, ethnicity, MS type	Medication history (reason for drug withdrawal)	TTE* (TTE as reported in the article whether not clearly specified)	Symptoms’ description	Action taken to manage the adverse event	Adverse event’s outcome
Alemtuzumab (67)	58, F, Caucasian, RRMS	IFN-β-1a (lack of efficacy) and corticosteroids	2 days	Petechiae and epistaxis, Hb 13.8 g/dL, WBC 7100/μL, platelet 89×10^3^/ μL	Posterior nasal packing	Resolution
Alemtuzumab (43)	47, F, Caucasian, RRMS	IFN-β-1a, fingolimod and natalizumab (disease’s progression)	23 days	High fever and severe neutropenia (WBC 1750/μL; ANC, 40/μL; lymphocytes 1,650/μL; PLT 13,000/Μl), which resulted in septic shock by *S. aureus* and ARDS	Intravenous vancomycin, ceftriaxone and gentamicin	Death
Alemtuzumab (40)	38, F, NA, RRMS	IFN-β-1a (clinical and radiological progression), natalizumab (JC virus positivity), GA (severe clinical relapses)	24 days	Severe leukopenia (WBC 0.93 × 10^3^/μL), neutropenia (0.83 × 10^3^/μL) and lymphopenia (0.04 × 10^3^/μL), with a platelets’ reduction (PTL: 140 × 10^3^/mL)	Antibiotic therapy	All events resolved, except lymphopenia
Alemtuzumab (48)	Early-20s, M, NA, RRMS	IFN-β-1a (failure), natalizumab JC virus positivity, fingolimod (severe rebound), methylprednisolone and plasma exchange	4 weeks	Severe neutropenia (WBC count, 600/μL; ANC, 470/μL)	GSF	Resolution
Alemtuzumab (42)	44, F, Caucasian, RRMS	IFN-β-1a, natalizumab (increased risk of PML), fingolimod (severe relapse and persistent magnetic resonance activity)	1 month	Grade 4 leukopenia and grade 3 neutropenia (WBC 850/μL, ANC 760/μL, ALC 10/μL)	Fluconazole, amoxicillin and acyclovir	Resolution
Cladribine (34)	49, F, NA, RRMS	GA (new lesions on MRI, right upper limb hyposthenia and paresthesia)	2 weeks	Neutropenia (WBC 1.38 × 1.000/mm^3^, neutrophils 0.30 × 1,000/μL, lymphocytes 0.70 × 1,000/μL)	Ciprofloxacin and filgrastim	Resolution, positive rechallange
Fingolimod (59)	71, F, NA, NA	NA	2 weeks	Lymphocyte counts decreased from 800 to 176 × 10^6^/l.	The drug administration was changed from daily to every other day	Resolution
Natalizumab (60)	50, F, NA, RRMS	IFN-β-1a (lack of efficacy) GA (local site reactions)	1 week	AIHA (Hb 5.4 g/dL, HCT 15.2%, serum bilirubin 3.31 mg/dL, serum unconjugated bilirubin 2.79 mg/dL, LDH 1590 UI/l, serum haptoglobin <5.5 mg/dL, positive DAT)	IVIG and corticosteroids	Improvement
Natalizumab (81)	25, F, African American, RRMS	IFN-β-1a (inadequate response)	3 weeks	Petechial lesions, ITP (platelet count dropped to 6,000/μL).	Prednisone	Resolution, positive rechallange
Ocrelizumab (72)	32, M, NA, NA	IFN, fingolimod (persistent lymphopenia), dimethyl fumarate (clinical relapse), rituximab (neutropenia)	1 month	Neutropenia (WBC 2.4 × 10^9^/μL, neutrophils 0.240 × 10^9^/μL)	G-CSF	Resolution
Siponimod (25)	55, M, NA, SPMS	IFN-β-1a (ongoing disease activity), fingolimod (enrollment in the Compassionate Use Program: “CBAF312A2001M” to receive siponimod)	1 month	Severe lymphopenia (200/mmc)	Siponimod dosage was changed from 2 mg/die to 1 mg every other day	Improvement

AIHA, Autoimmune hemolytic anemia; ANC, Absolute Neutrophil Count; ALC, Absolute Lymphocyte Count; ARDS, Acute Respiratory Distress Syndrome; DAT, Direct Antiglobulin Test; IFN, interferon; JC, John Cunningham; MRI: NA, Not Available; PLT, platelet; PML, Progressive multifocal leukoencephalopathy; WBC, White Blood Cell. ^*^TTE from the first DMT administration.

**Table 2:** Overview of DMTs-induced late-onset myelotoxicities.

DMT (ref)	Age (years), sex, ethnicity, MS type	Medication history (reason for drug withdrawal)	TTE* (TTE as reported in the article whether not clearly specified)	Symptoms’ description	Action taken to manage the adverse event	Adverse event’s outcome
Alemtuzumab (48)	Mid-20s, F, NA, RRMS	IFN-β-1a (failure), teriflunomide (failure), fingolimod (radiologic and clinical disease activity persisted)	6 weeks	Febrile neutropenia (WBC, 1000/μL; ANC, 300/μL) and sinusitis	Intravenous antibiotics and GSF	Resolution, positive rechallange
Alemtuzumab (41)	34, F, NA, RRMS	GA and natalizumab	70 days	Neutropenia (500 neutrophils/μL) with virtual absence of B-cells (0.6% of total lymphocytes), low values of CD4-T-cells (6.6%) and predominance of CD8-T-cells (48%) and NK-cells (47%)	Low-dose corticosteroids	Improvement
Alemtuzumab (33)	52, M, middle eastern ethnicity, RRMS	IFN-β-1a (relapse), natalizumab (positive JC virus antibody), fingolimod (relapse of lower limb weakness and severe lymphopenia)	8 months	AIHA, alveolar hemorrhage, nephropathy and stroke	Methylprednisolone, plasma exchange and intermittent hemodialysis	Resolution
Alemtuzumab (78)	28, M, Caucasian, RRMS	IFN, fingolimod (lymphocytopenia)	9 months	Thrombocytopenia (platelet count 11×10^9^/L) and immune mediated thyroid disease	IVIG and prednisolone	Improvement
Alemtuzumab (44)	34, M, NA, RRMS	IFN-β-1a (relapse), natalizumab (positive JC virus antibody), fingolimod (relapse)	11 months	Asthenia and palpitations, severe anemia (Hb 5.2 g/dL). The diagnosis was AIHA (serum haptoglobin: <8 mg/dL, LDH 1114 UI/L)	Methylprednisolone, IVIG and transfusion of erythrocytes	Resolution
Alemtuzumab (45)	28, M, NA, RRMS	IFN beta, mitoxantrone, fingolimod (relapse)	11 months	Mild jaundice, tachycardia, tachypnea. The diagnosis was AIHA (Hb 4.8 g/dL, decreases during the hospitalization to 3.5 g/dL)	Methylprednisolone, IVIG and rituximab	Improvement
Alemtuzumab (78)	26, M, Caucasian, RRMS	IFN, glatiramer acetate, fingolimod (marked lymphocytopenia)	11 months	Petechial bleeding, severe thrombocytopenia (platelet count 2×10^9^/L), minor hemolytic anemia (Hb 12.8 g/dL) and immune mediated thyroid disease. The diagnosis was ITP	Corticosteroids and IVIG	Resolution
Alemtuzumab (35)	31, NA, Caucasian, MS	IFNβ-1α, natalizumab (JC virus positivity), fingolimod (sustained B-cell lymphopenia)	1 year	AIHA (HCT 19.0%, Hb 7.8 g/dL, hemoglobinuria, RBC agglutination on peripheral blood smear and positive direct Coombs test)	Corticosteroids, rituximab, plasmapheresis, and RBC transfusions	Resolution
Alemtuzumab (69)	42, F, NA, NA	IFN, glatiramer acetate, dimethyl fumarate	13 months (first cycle and second cycle performed in November 2019 and 2020, respectively)	Abnormal complete blood count (Hb 10.2 g/dL, RBC 4.65×10^12^/L, HCT 32.7%, WBC 0.79×10^9^/L, ANC 0.00×10^9^/L, platelet count 28×10^9^/L), fever, tachycardia (119 beats/min). The diagnosis was myelodysplastic syndrome	G-CSF and corticosteroid	Temporary improvement
Alemtuzumab (21)	Mid-30s, F, RRMS	IFN-β-1a (relapses), mitoxantrone, natalizumab, GA, fingolimod (severe relapse)	18 months	Refractory immune thrombocytopenia, vasculitis, myelofibrosis and Guillain-Barré syndrome (the diagnosis was secondary immune disease)	Prednisone, IVIG, rituximab, cyclophosphamide and eltrombopag	Resolution
Alemtuzumab (31)	35, M, NA, RRMS	Methylprednisolone, natalizumab (JC antibody positivity), GA (side effects)	2 years	Cardiac sarcoidosis, mild microcytic anemia and mild leukopenia and immune thrombocytopenia (platelet count of <5,000)	Dexamethasone and IVIG	Resolution
Alemtuzumab (27)	45, M, NA, RRMS	IFN-β (neutralizing antibodies), fingolimod (elevations in liver transaminases), DMF (JC virus seropositivity), IVIG	28 months	Neutropenia and thrombocytopenia (ANC 70/mL and platelet count 23,000/mL)	Steroids	Unchanged after 4 months
Alemtuzumab (64)	39, M, NA, RRMS	IFN-β-1a, natalizumab, fingolimod, dimethyl fumarate	45 months	Immune-mediated thrombotic thrombocytopenic purpura [severe thrombocytopenia (5×10^9^ cells/L), anemia (8.5 g/dL), elevated inflammatory markers (CRP 42 mg/ L and ferritin 1,186 μg/L)]	Plasma exchange, corticosteroids and caplacizumab	Resolution
Alemtuzumab (74)	39, F, NA, RRMS	Glatiramer acetate and IFN (clinical and radiological disease activity)	Unclear (two months following the third dose of alemtuzumab)	Persistent and progressive anemia, abnormally low reticulocyte count and elevated haptoglobin.	Not reported	Resolution
Alemtuzumab (21)	28, F, NA, RRMS	Methylprednisolone	Unclear (several weeks after commencing the initial treatment)	Red cell aplasia, immune thrombocytopenia and immune neutropenia	Steroids, IVIG, rituximab, red cell transfusions, vincristine, G-CSF, cyclosporine and mycophenolate	Improvement
DMF (46)	36, F, NA, RRMS	IFN-β-1a	4 months	Lymphopenia (WBC 3.6 × 10^9^ L, ALC 0.4 × 10^9^/L).	Methylprednisolone and iron supplementation	Resolution, positive rechallange
DMF (30)	50, F, Caucasian, MS	IFN-β-1b (side effects), GA (patient no longer willing to self-inject)	6 months	Grade III lymphopenia (ALC 0.43×10^9^/L)	DMF withdrawal	Unchanged
DMF (76)	38, F, NA, MS	NA	3 years	Severe hemolytic anemia and reactive leukocytosis (RBC 1.37 × 10^12^/L; HCT, 14.7%; Hb, 4.7 g/dL; reticulocytes, 13.34%; WBC 13.3 × 10^9^/L; total bilirubin, 3.07 mg/dL; haptoglobin, 1 mg/dL; LDH 756 U/L)	Prednisone	Resolution
Fingolimod (59)	28, F, NA, NA	NA	5 weeks	Lymphocyte count decreased (from 1,292 × 10^6^/l to 244 × 10^6^/l)	The drug administration was changed from daily (0.5 mg/day: 3 mg/6 days) to 5 times/6 days	Resolution
Fingolimod (59)	35, F, NA, NA	NA	7 weeks	Neutropenia (ANC 1.3 × 10^9^/L)	The drug administration was changed to 4 times/6 days (0.5 mg/day: 2.0 mg/6 days)	Resolution
Fingolimod (56)	36, F, Caucasian, RRMS	IFN beta (lack of efficacy, anti-JCV antibodies)	2 months	Leukopenia (2.72 × 10^9^/L) and lymphopenia (0.140 × 10^9^/L)	Fingolimod withdrawal	Resolution
Fingolimod (47)	59, F, NA, NA	IFN-β (persistent lymphopenia and neutropenia)	2 months	Severe thrombocytopenia (platelet count 1 × 10^9^/L). The diagnosis was ITP	Prednisolone, IVIG, azathioprine and hydroxychloroquine.	Resolution
Fingolimod (57)	19, NA, NA, NA	–	10 months	Fever, jaundice, nausea and fatigue, AIHA (Hb 6.0 g/dL, serum bilirubin 2.3 mg/dL serum unconjugated bilirubin 0.84 mg/dL, LDH 537 UI/l, serum haptoglobin <5 mg/dL). DAT was positive	Blood transfusion and methylprednisolone treatment, fingolimod discontinuation and corticosteroids	Improvement
Fingolimod (47)	22, F, NA, RRMS	DMF (lymphopenia)	1 year	Severe thrombocytopenia (platelet count 12 × 10^9^/L). The diagnosis was ITP	Prednisolone	Resolution
Fingolimod (51)	32, F, NA, RRMS	Immunomodulators	18 months	Lymphopenia (lymphocytic count <200/μl)	Fingolimod withdrawal	Resolution
Fingolimod (47)	51, F, NA, NA	IFN-β	19 months	Severe thrombocytopenia (platelet count 4 × 10^9^/L). The diagnosis was ITP	Prednisolone, IVIG, azathioprine, hydroxychloroquine, eltrombopag and romiplostim fingolimod discontinuation	Resolution
Fingolimod (20)	43, F, NA, RRMS	Mitoxantrone, IFN beta-1a (disease’s worsening)	2 years	AIHA (RBC 1.64, Hb 5.3, MCV 120.9, RDW 17.8, % neutrophils 89.3, % lymphocytes 5.1, % monocytes 3.8, reticulocyte count 29.8, corrected reticulocyte count: 5.3%, BUN: 13.6 mg/dL). DAT was positive	Fingolimod discontinuation	Resolution
Fingolimod (75)	32, F, Caucasian, RRMS	–	4 years	Thrombocytopenia (platelet count 5×10^9^/L). The diagnosis was probable drug –induced immune thrombocytopenia	Platelet transfusion, IVIG, corticosteroids and fingolimod discontinuation	Resolution
Glatiramer acetate and IFN-β 1a (22)	65, F, NA, RRMS	Corticosteroids	4 months (GA), 1 month (IFN)	Severe lymphopenia (lymphocyte 200 /μl). The diagnosis was tuberculous lymphadenitis	IFN-β 1a discontinuation, anti-tuberculosis treatment	Resolution
Glatiramer acetate (79)	40, F, NA, NA	IFN	2 months	Fever, petechial rash on legs, Hb 12.1 g/dL leucocyte count 4,870/mm3, platelet count 1,000/mm3, LDH 515 U/L (diagnosis of refractory symptomatic ITP)	Glatiramer acetate withdrawal, tranexamic acid, corticosteroid and IVIG, splenectomy	Improvement
IFN β (58)	31, F, Caucasian, RRMS	Methylprednisolone	9 weeks	Neutropenia (neutrophil count 0.55 × 10^9^/l).	IFN β discontinuation	Positive rechallange
IFN-β-1a (76)	22, F, NA, RRMS	Methylprednisolone	6 months	Purpuric rash, ITP (WBC 8800 U/μL, platelet 6,000/μL)	IFN withdrawal, methylprednisolone and IVIG	Resolution
IFN β-1b (61)	26, F, NA, RRMS	Prednisolone	11 months	Icter and fatigue. Laboratory tests showed anemia, indirect hyperbilirubinemia (Total bilirubin: 8.5 mg/dL, direct bilirubin: 0.4 mg/dL, LDH: 819 IU/L, indirect coomb’s test: positive, Direct coomb’s test: Positive). The diagnosis was AIHA	IFN discontinuation	Resolution
IFN β-1a (63)	42, F, African-American, NA	NA	1 year	Weakness, Hb and HCT levels were 3.8 g/dL and 12%, respectively, a reticulocyte count of 1.8%, a platelet count of 10 K/mL, and a WBC count of 1.9 K/mL. Serum ferritin was 22 ng/mL. The diagnosis was aplastic anemia	Antithymocyte globulin, cyclosporine and prednisone. IFN withdrawal	Improvement
IFN (83)	31, M, NA, RRMS	No medication history	2 years	Hb 4.4 g/dL, reticulocyte percentage 6.3% (0.5–1.5), lactic dehydrogenase 499 U/L (95–213), total bilirubin 2.40 mg/dL (0.2–1.6), direct and indirect Coombs’ tests were positive, the potential etiologies of AIHA were ruled out.	IFN discontinuation, corticosteroids	Resolution
IFN β-1a (82)	22, M, NA, RRMS	IFN-β-1b	1 year	ITP	Not reported	Not reported
IFN β (62)	35, F, NA, NA	Methyl prednisone	2 years	Decreased WBC, Hb and platelet levels (WBC 2900 dL, Hb 9.9 g/dL, platelet 168,000/dl). The diagnosis was dysplastic hematopoiesis	IFN discontinuation, conservative therapy	Resolution
IFN β (62)	39, F, NA, NA	Prednisone	5 years	Reduction of WBC, Hb and platelets (WBC: 3000/dl, Hb: 9.8 g/dL, PLT: 151.000), dysplastic hematopoiesis	IFN β discontinuation, conservative therapy	Resolution
IFN β-1a (19)	52, F, NA, RRMS	NA	10 years	Limb and oral mucosa petechiae and hematochezia, severe thrombocytopenia (13 G/L). The diagnosis was ITP	IFN discontinuation, tranexamic acid, corticosteroids	Positive rechallange
IFN β-1b (77)	25, F, NA, NA	NA	NA	Comatose state, decreased RBC, Hb and HCT. Platelet count: 60.000/mm^3^. LDH, AST and ALT levels were 10, 80 and 100 times higher the normal range. The diagnosis was thrombotic thrombocytopenic purpura and hemolytic uremic syndrome	Plasmapheresis, corticosteroids and IFN withdrawal	Resolution
Natalizumab (66)	35. F. NA, RRMS	IFN-β-1a (increased disability and clinical relapse), rituximab (right lower limb weakness)	7 weeks (3 weeks after receiving the second course of natalizumab)	Thrombocytopenia (petechiae and purpuric skin rashes, severe low platelet count - below 5,000)	Dexamethasone, natalizumab discontinuation, corticosteroids	Resolution
Natalizumab (54)	52, F, NA, RRMS	IFN β-1a and GA (side effects)	12 weeks (after the third treatment with natalizumab)	Acute infusion reaction, whole-body purpura, platelet count of 43.000/mm^3^. Platelet antibodies to platelet-specific antigens positive. Antibodies against natalizumab positive. The diagnosis was thrombotic thrombocytopenic purpura	Natalizumab discontinuation, methylprednisolone	Improvement
Natalizumab (37)	40, M, Caucasian, RRMS	Azathioprine (progression of lesional load), GA (lack of efficacy), fingolimod (alteration in liver function), DMF (lack of efficacy)	23 weeks (3 weeks after the 5th Natalizumab-administration)	Petechiae, severe thrombocytopenia (0 PLT/mm3). The diagnosis was acute immune thrombocytopenia	Natalizumab discontinuation, methylprednisolone	Improvement
Natalizumab (60)	61, F, NA, RRMS	NA	10 months (3 weeks after the 10th natalizumab infusion)	Severe thrombocytopenia (2000 platelets/mm3) and hemorrhagic diathesis (diagnosis of ITP)	Prednisone and IVIG	Improvement
Natalizumab (52)	33, M, NA, RRMS	IFN-β-1a (clinical and radiologic disease reactivation)	15 months (after 15 infusions)	Severe anemia (Hb 8.4 g/dL)	Natalizumab withdrawal, PRCU, intravenous alfaepoetin, iron therapy and prednisone	Resolution
Natalizumab (80)	49, F, NA, RRMS	No prior significant medical history	16 months	Fatigue, exercise intolerance, macrocytic anemia (Hb 7.4 g/dL)	Blood transfusion and natalizumab discontinuation	Resolution
Natalizumab (52)	38, F, NA, RRMS	IFN-β-1a and mitoxantrone (infectious complication)	16 months	Severe symptomatic anemia (Hb 7.5 g/dL)	PRCU, natalizumab discontinuation, B group vitamin, and folic acid	Resolution
Natalizumab (53)	51, F, NA, RRMS	Azathioprine, IFN-β-1a	3 years	Severe anemia (Hb 7.3 g/dL) and a blood reticulocyte rate at the lower limit of normal (0.020 mL/mcL, normal 0.018–0.114 mL/mcL, and 0.66%, normal 0.38–2.13%)	Natalizumab discontinuation, blood transfusion	Resolution
Natalizumab (73)	21, NA, NA, RRMS	IFN (high disease activity)	4 years	Hb 8 g/dL, WBC 1.42×10^9^/L, lymphocyte count 0.11 ×10^9^/L	Natalizumab withdrawn	Improvement
Ocrelizumab (28)	34, M, NA, PPMS	NA	42 days	Neutropenia (ANC 0.5 × 10^9^), fever, abdominal tenderness (diagnosis of neutropenic enterocolitis)	Broad-spectrum intravenous antibiotics and G-CSF	Resolution
Ocrelizumab (26)	16, F, NA, RRMS	IFN-β-1a and GA (disease’s worsening), natalizumab (JC virus seropositivity), fingolimod (lack of efficacy)	7 weeks	Fever, severe leukopenia (WBC 1.67 × 10^3^/μL), neutropenia (ANC 0.7 × 10^3^/μL) and lymphopenia (0.7 × 10^3^/μL)	Ciprofloxacin and filgrastim	Improvement
Ocrelizumab (39)	35, F, NA, RRMS	GA, IFN-β-1a (side effects), DMF	3 months	Fatigue, myalgia, fever, mucositis, neutropenia (ANC 0 × 10^9^/L) and lymphopenia (ALC 0.3 × 10^9^/L)	Cefepime and acyclovir, filgrastim and methylprednisolone	Improvement
Ocrelizumab (32)	21, F, NA, RRMS	Methylprednisolone, DMF (relapses), rituximab	4 months	Grade IV neutropenia (ANC 0.1 × 10^9^/L).	Lidaprim, acyclovir and ocrelizumab discontinuation	Improvement
Ocrelizumab (17)	44, F, NA, PPMS	NA	7 months	Febrile neutropenia, secondary herpetic stomatitis and gangrenous ecthyma (ALC 0.34 × 10^3^/μl and ANC 0.04 × 10^3^/μl)	Cefepime, acyclovir and voriconazol	Improvement
Ocrelizumab (68)	35, F, NA, RRMS	NA	7 months	Asymptomatic agranulocytosis (neutrophil: 20/mm3)	None	Spontaneous resolution
Ocrelizumab (20)	41, M, NA, RRMS	DMF (clinical and radiological worsening)	7 months (1 month after the second dose)	Severe neutropenia (ANC 0.3 × 10^3^/μL)	G-CSF	Resolution
Ocrelizumab (36)	26, F, NA, RRMS	NA	10 months	Fever, WBC 1.1 × 10^9^/L, ALC 0.3 × 10^9^/L, ANC 0 × 10^9^/L and AMC 0.8 × 10^9^/L, CRP 36 U/L and procalcitonin 1.0 U/L.	Acyclovir and ceftriaxone	Resolution
Ocrelizumab (70)	40, F, Malay, RRMS	Natalizumab (relapse)	10 months (14 weeks after the third ocrelizumab infusion)	Bilateral blurring vision, worsening of weakness, fever, WBC 2.3×10^9^ cells/L and ANC 0.11×10^9^ cells/L. ANC worsened to 0.02×10^9^ cells/L	Intravenous antibiotics	Resolution
Ocrelizumab (20)	41, F, RRMS	IFN-β-1a (disease’s worsening), natalizumab (JC positivity and desire for pregnancy), fingolimod (radiological disease activity)	1 year (5 month after the second dose)	Severe neutropenia (ANC 0.08 × 10^3^/μL)	G-CSF	Resolution
Ocrelizumab (68)	28, M, NA, RRMS	Dimethyl fumarate (persistence of clinical and radiological activity)	19 months and 2 weeks (eight weeks after the fourth ocrelizumab infusion)	Severe neutropenia (neutrophils: 322/mm^3^)	Antibiotic prophylaxis	Resolution, positive rechallange
Ocrelizumab (65)	33, F, NA, RRMS	Alemtuzumab (disease resistance)	2 years (4 days after receiving 4 full doses)	Neutropenia (ANC: 0), fever and mouth ulcers	G-CSF	Resolution
Ocrelizumab (65)	47, F, NA, NA	Natalizumab and fingolimod	2 years (6 months after three doses)	Neutropenia (ANC: 0) and fever	G-CSF, cefepime and ocrelizumab discontinuation	Resolution
Ocrelizumab (65)	59, F, NA, SPMS	NA	2 years	Fever, ANC of 0.01 and WBC of 1.70 × 109 cells/L	Piperacillin and tazobactam, amoxicillin/clavulanate	Resolution
Ocrelizumab (68)	38, F, NA, RRMS	Natalizumab (JC virus seroconversion) and fingolimod (disease activity)	25 months	Stomatitis, fatigue and febrile agranulocytosis (ANC 30/mm3)	G-CSF	Resolution, positive rechallange
Ocrelizumab (71)	56, M, NA, RRMS	NA	26 months (10 weeks after the last dose of ocrelizumab which as administered 6-monthly for 2 years)	Severe neutropenia (0.2×10^9^/L), mild lymphopenia (0.8×10^9^/L)	Antibiotics, filgrastim and ocrelizumab discontinuation	Resolution
Ocrelizumab (65)	35, M, NA, RRMS	NA	2 years and 5 months (5 months after receiving 4 full doses)	Neutropenia (ANC: 0.005), fever and mucosal ulcers	Vancomycin, meropenem and acyclovir	Resolution
Ocrelizumab (21)	51, M, NA, SPMS	IFN-β-1a (disease’s worsening), natalizumab (JC virus index positivity), fingolimod (disease’s worsening)	2 years and 8 months (75 days after the last infusion)	Neutropenia (ANC 1.10 × 103/μL), stomatitis and fever	Antibiotics	Improvement
Ocrelizumab (30)	38, M, NA, PPMS	NA	3.5 years (3 months after last drug infusion)	Severe neutropenia (ANC of 0.0 × 10^9^/L and ALC of 0.8 × 10^9^/L)	Broad-spectrum intravenous antibiotics, acyclovir, and filgrastim	Improvement
Ocrelizumab (65)	37, F, NA, RRMS	NA	5 years (the patient has been treated 6 monthly since 2017 until the development of neutropenia in January 2022)	Neutrophil count of 0.94 ×10^9^ cells/L	Not reported	
Ocrelizumab (65)	36, F, NA, RRMS	NA	One month and half after the last dose of ocrelizumab	Fevers, mouth ulcers, mucositis, headache, neutropenia	Piperacillin, tazobactam and G-CSF	Not reported
Ocrelizumab (65)	29, NA, NA, RRMS	Natalizumab, fingolimod	Five months after the last dose of ocrelizumab	Fever, abdominal pain, neutropenia	G-CSF	Not reported
Ocrelizumab (65)	35, NA, NA, RRMS	NA	NA	Fever, mouth ulcers, headache, urinary urgency, neutropenia	Ceftriaxone, acyclovir, then amoxicillin/clavulanate and G-CSF, ocrelizumab withdrawal	Resolution
Ocrelizumab (65)	28, NA, NA, RRMS	NA	NA	Neutropenia (ANC 0.05 × 10^9^ cells/L)	G-CSF and the treatment with ocrelizumab was stretched to 7-monthly infusions	Not reported

AIHA, Autoimmune hemolytic anemia; ANC, Absolute Neutrophil Count; ALC, Absolute Lymphocyte Count; DAT, Direct antiglobulin test; Hb, hemoglobin; HCT, hematocrit; IFN, interferon; ITP, immune thrombocytopenic purpura; GA, glatiramer acetate; IVIG, intravenous immunoglobulin; JC, John Cunningham; LDH, serum lactate dehydrogenase; NA, information not available; PLT, platelet; PML, Progressive multifocal leukoencephalopathy; PRCU, packed red blood cell units; RBC, red blood cells; WBC, white blood cell. ^*^TTE from the first DMT administration.

**Table 3:** Case reports describing the occurrence of myelotoxicities in newborns from mothers who received natalizumab during pregnancy.

Author (ref)	Brief case description
Godano (29)	A woman had received natalizumab during pregnancy every 6 weeks. The baby was born at 37 + 5/7 weeks of gestation. The baby’s weight was 2,915 g, the APGAR score at 1 and 5 min was 9/10; globally, the newborn underwent a regular adaptation to extra-uterine life. Blood exams performed after birth revealed a low-moderate anemia (Hb: 10.9 g/dL; hematocrit 34.1%). Blood samples collected at 40 days of life showed the Hb was 8 g/dL and the hematocrit was 23.5%. The baby received therapy with erythropoietin (EPO) and at 4 months of life, the Hb was 12 g/dL and the hematocrit was 37.4%
Guilloton (49)	A 27-year-old Caucasian woman began her third pregnancy after 3 injections of natalizumab. The patient decided to continue this treatment during the pregnancy. The baby (whose ethnic origin was Caucasian and black African), was born 2 weeks before the term. The weight was 3,140 g and the APGAR score at 10/10. Blood exams performed 12 days after birth revealed a mild pancytopenia with leucopenia, thrombocytopenia and anemia (Hb 9.3 g/dL, hematocrit 26%, blood platelets 126.000). Blood exams normalized at 3 months of age
Ciron (50)	A 28-year-old woman became pregnant while she was receiving natalizumab. She gave birth to a healthy boy at 40 weeks and 4 days’ gestation. The newborn had a low platelet count at birth (124×10^9^ /l, normal ranging from 150 to 400) without any other hematological abnormalities. Blood count was normal on analysis 10 days later
Haghikia (55)	This case series described the occurrence of hematological abnormalities, including anemia and thrombocytopenia, among 10 infants whose mothers had received 1–9 natalizumab infusions during pregnancy. For these newborns, the mean gestational age at birth was 38.4 weeks while the mean birth weight was 2,723 g

The publication date of the included studies ranged from 2006 to 2023. The results of risk of bias assessment of included studies are shown in Supplementary Tables S2, S3 (for case reports and case series, respectively). Neonatal cases (29, 49, 50, 55) were not included in the risk of bias assessment. Fifty-three case reports and 10 case series were evaluated using the risk of bias assessment tool. Regarding case reports, 8 studies (33, 37, 42, 58, 67, 70, 75, 81) had the highest methodological quality, together with 3 other studies (30, 43, 56) that similarly reported high quality information. For the remaining case reports, the risk of bias evaluation revealed mainly the lack of data on patients’ race and patients’ history presented as timeline. Regarding the risk of bias assessment for case series, we found that out of 8 studies, only one (20) had a high methodological quality; for the remaining studies the lack of data mainly concerned the clear identification of inclusion/exclusion criteria, the consecutive and complete inclusion of patients and the reporting of presenting sites and demographic information.

The main characteristics of included case reports and case series are reported in Tables 1, 2, which describe early-onset (events that occurred within 1 month after the first administration of the DMT) and late-onset (events that occurred after the first month of therapy) hematological disorders, respectively. Cases related to newborns are described in Table 3. An overview of the number of hematological disorders by DMTs is reported in Table 4. Lastly, the description of included case reports and series is presented hereafter by suspected drugs.

**Table 4:** Overview of hematological disorders by suspected DMTs.

Main myeloid-lymphoid ADR	ALE	CLA	DMF	FIN	IFN	GA	NAT	OCR	SIP	Total ADRs
AIHA	4	–	–	2	2	–	1	–	–	9
Anemia (including aplastic and hemolytic)	1	–	1	–	1	–	4	–	–	7
Dysplastic hematopoiesis	–	–	–	–	2	–	–	–	–	2
Immune thrombocytopenia	4	–	–	4	3	–	3	–	–	14
Leukopenia,lymphopenia and/or neutropenia	5	–	–	1	–	–	1	4	–	11
Lymphopenia	–	–	2	3	–	1	–	–	1	7
Neutropenia	4	1	–	1	1	–	–	21	–	28
Thrombocytopenia	2	–	–	–	–	1	1	–	–	4
Thrombotic thrombocytopenic purpura	–	–	–	–	1	–	1	–	–	2
Total ADRs/DMT	20	1	3	11	10	2	11	25	1	84

*ALE, alemtuzumab; CLA, cladribine; DMF, dymethil fumarate; FIN, fingolimod; IFN, interferon; IFN/GA, interferon/glatiramer acetate; NAT, natalizumab; OCR, ocrelizumab; SIP, siponimod.

### 3.1 Alemtuzumab-induced hematological disorders

Sixteen case reports (21, 24, 27, 31, 33, 35, 40–45, 64, 67, 69, 74) and two case series (48, 77) described the occurrence of hematological disorders after alemtuzumab treatment. Of these studies, 5 concerned the occurrence of early-onset toxicities (40, 42, 43, 48, 67), including severe cases of leukopenia, neutropenia, lymphopenia and thrombocytopenia that occurred in four female and one male patients, aged <58 years, with a medication history that included at least IFN and/or natalizumab for all of them. Apart from one case that resulted in patient’s death (43), the remaining ones described the occurrence of hematological toxicities resulted in a full recovery. Alemtuzumab-induced late-onset hematological disorders were instead described in the remaining case reports and series (24, 27, 31, 33, 35, 41, 44, 45, 64, 69, 77). These events occurred in 8 male and 4 female patients [in one case (35) the sex was not reported]. The medication history included different therapies, mainly IFN, natalizumab and fingolimod for the majority of patients. The TTE ranged from 6 weeks to 45 months. ADRs reported were varied, with cases reporting AIHA, neutropenia, anemia and immune thrombocytopenia. In the majority of cases, the treatment with corticosteroids, intravenous immunoglobulins (IVIG) and transfusion led to improvements in bone marrow function. Neither of the reported cases had a fatal outcome.

Lastly, for two cases (21, 74) the time of hematological toxicity occurrence was unclear. Both cases concerned female patients aged <40 years, diagnosed with RRMS, who experienced cases of anemia and thrombocytopenia.

### 3.2 Natalizumab-induced hematological disorders

Nine studies described the occurrence of natalizumab-induced hematological disorders (37, 52–54, 60, 66, 73, 80, 81), of which 2 were case series (52, 60). Two cases of early onset hematological toxicity were found in the literature, of which one (60) concerning a 50-year-old female patient who experienced AIHA only 1 week after the first drug administration. The prompt treatment with IVIG and corticosteroids led to patient’s improvement after 2 months. The other case (81) concerned, instead, a 25-years-old African American woman who experienced ITP 3 weeks after the drug administration. Although the event resolved at the beginning, it re-occurred with the second infusion of the drug. The other cases were related to the occurrence of late-onset hematological toxicities (for which the TTE ranged from 12 weeks to 4 years), occurring in 5 female patients and 2 male patients aged <61 years. In one case (73) the patient’ sex was not specified. These cases were mainly related to the occurrence of severe anemia and thrombocytopenia (including autoimmune thrombocytopenia and thrombocytopenic purpura), and the majority with a progressive resolution after treatment with steroids and transfusion.

We also found 4 articles describing the occurrence of myelotoxicities in newborn from mothers who received natalizumab during pregnancy (29, 49, 50, 55). These cases were not included in the risk of bias assessment. Godano E et al. reported the case of a woman who had received natalizumab during pregnancy every 6 weeks. The baby was born at 37 + 5/7 weeks of gestation and blood tests showed the Hb was 8 g/dL and the hematocrit was 23.5%. The baby received therapy with erythropoietin (EPO) experiencing improvements of blood exams at 4 months of life (29). Another case was reported by Guilloton et al. who described the case of a 27-year-old woman who received the drug during pregnancy. The baby, was born 2 weeks before the term and blood exams performed 12 days after birth revealed a mild pancytopenia with leucopenia, thrombocytopenia and anemia. Blood exams normalized at 3 months of age (49). Ciron et al. reported the case of a 28-year-old woman who became pregnant while she was receiving natalizumab. The baby, was born at 40 weeks and 4 days of gestation. The newborn had a low platelet count at birth and experienced a improvement 10 days later (50). Lastly, Haghikia et al. described hematological abnormalities, including anemia and thrombocytopenia, among 10 infants whose mothers had received 1–9 natalizumab infusions during pregnancy (55). A detailed description of cases involving newborns is reported in Table 3.

### 3.3 Ocrelizumab-induced hematological disorders

Ten case reports (17, 23, 26, 28, 32, 37, 39, 70–72) and three case series (21, 65, 68) concerned the occurrence of hematological toxicities in patients receiving ocrelizumab. Apart from the case reported by Marrodan et al. (72) who described the occurrence of early onset neutropenia in a 32-year-old man, the remaining cases were related to late onset events with a TTE that ranged from 42 days to 5 years. Interferons and DMF mainly represented the medication history of these patients. Patients were aged <56 years and they presented multiple signs and symptoms that mainly included severe neutropenia. The antibiotic and antiviral prophylaxis together with G-CSF led to patients’ recovery in the majority of cases. For 4 cases described by Pang et al. (65) clinical and demographic data, including those related to the time to event, patients’ sex and medication history, were missing.

### 3.4 Interferon β-induced hematological disorders

Eight case reports (18, 58, 61, 63, 76, 77, 82, 83) and one case series (62) reporting the association IFN β-myeloid toxicity were found in the literature. Apart from one case (77) for which the time of toxicity occurrence was not inferable, all cases were related to late-onset events, occurring from 9 weeks to 10 years after the first drug administration. The majority of patients were female, younger than 52 years, with a medication history completely absent or mainly consisting in corticosteroids. The events mainly reported were neutropenia, anemia and thrombocytopenia. The treatment with conservative therapy and corticosteroids together with IFN β withdrawal led to full recovery in all patients.

### 3.5 Fingolimod-induced hematological disorders

Overall, five case reports (19, 51, 56, 57, 75) and 2 case series (47, 59) reported the occurrence of hematological toxicities following the treatment with fingolimod. Among cases reported by Tanaka et al. (59) there was one a case of lymphopenia that occurred 2 weeks after the beginning of fingolimod therapy in a 71-year-old woman that resolved after changing the frequency of the drug administration. The other cases reported in this case series were instead related to the occurrence of late-onset lymphopenia and neutropenia occurred after 5 weeks and 7 weeks, respectively, in 2 young women. For the remaining studies the TTE ranged from 2 months to 4 years. These cases mainly concerned cases of lymphopenia and AIHA. For the majority of cases fingolimod discontinuation and treatment with corticosteroids led to complete recovery of blood exams.

### 3.6 Dimethyl fumarate-induced hematological disorders

Three case reports (30, 38, 46) described the occurrence of late-onset hematological disorders (TTE ranged from 4 months to 3 years) in 3 women aged <50 years. In one case the medication history was not reported (38), while in the remaining cases patients had already received before IFN and glatiramer acetate. Anemia and lymphopenia were the events mainly reported. Only in one case (30) lymphopenia persisted for over 5 years despite treatment discontinuation. In their article, Zecca et al. (46) also reported that, after the review of medical records of their tertiary MS Centers (Lugano, Milan and Locarno), other 7 patients treated with DMF had discontinued because of ALC <0.5 × 109/L. These 7 cases were not considered for this review because of the lack of demographic, clinical and laboratory findings for the majority of them.

### 3.7 Other DMTs-induced hematological disorders

Finally, four case reports concerned the occurrence of hematological disorders following treatment with cladribine (34), siponimod (25), glatiramer acetate (79) and the combined therapy IFN/glatiramer acetate (22).

Maniscalco et al. reported a case of early non febrile neutropenia that occurred in 49-year-old RRMS female patient 2 weeks after the first cladribine cycle. The patient received ciprofloxacin for 5 days and filgrastim for 10 days with improvements in blood exams. After the second cladribine cycle, neutropenia occurred again, requesting a new cycle of ciprofloxacin and filgrastim with positive outcome (34).

Sparaco M et al. reported the case of a 55-year-old man who received siponimod in the context of the Compassionate Use Program: “CBAF312A2001M.” One month after the starting of the therapy, the patient developed severe lymphopenia (200/mmc). Siponimod dosage was changed from 2 to 1 mg/die with persistent lymphopenia (200/mmc) 1 month later, confirmed after another week. The drug was then administered at 1 mg every other day and 4 weeks later the lymphocyte count increased to 500/mmc (25).

Sagy I et al. described the occurrence of refractory symptomatic ITP in a 40-year-old woman 2 months after the initiation of therapy with glatiramer acetate. The condition was successfully managed by splenectomy (79).

The last case concerned a 65-year-old woman who developed mild to severe lymphopenia after having received glatiramer acetate and later IFN-β 1a. The patient started the treatment with glatiramer acetate in March 2007 and after 4 months her lymphocyte count decreased to 860 /μl. Four years later, IFN-β 1a was introduced and after 1 month the patient developed lymphopenia (22).

## 4 Discussion and conclusion

We carried out an extensive systematic review of studies published until January 5th 2024 on the Medline and Embase databases with the aim to provide an overview of case reports and series describing the occurrence of hematological disorders during the treatment with DMTs in patients with MS. We have reported data from 56 case reports and 11 case series concerning 84 adult patients who experienced myeloid toxicity during the treatment with a DMT and 13 newborns who developed this kind of toxicity due to a maternal exposure to a DMT. Out of 84 adults, 55 were female and 68 were younger than 50-year-old. When reported, RRSM was the most common form of MS. These demographic characteristics were expected considering that MS is a disease that worldwide affects more women than men (the prevalence ratio of MS of women to men is 2.3–3.5:1) and that the highest MS prevalence is in the age group 35–64 years (84–86).

Among myeloid toxicities, neutropenia was the most commonly reported, especially among patients treated with ocrelizumab. AIHA was frequently reported as well, mainly during the treatment with alemtuzumab. Overall, the DMTs most commonly reported as suspected in included case reports and series were alemtuzumab, natalizumab, ocrelizumab, IFN and fingolimod.

With regard to alemtuzumab, a risk of severe neutropenia and thrombocytopenia was already highlighted by the European Medicines Agency (EMA) at the end of a safety review carried out by the Pharmacovigilance Risk Assessment Committee at the request of the European Commission, under Article 20 of Regulation (EC) No 726/2004 (87, 88). In these communications, the EMA also reported that although rare, some ADRs, including thrombocytopenia, may occur 1 to 3 days of alemtuzumab infusion, while other events, mainly those autoimmune events, such as immune thrombocytopenic purpura, can occur within 48 months or longer after the last dose of alemtuzumab. With regard to the mechanisms underlying the occurrence of these events, thrombocytopenia might be the consequence both of a cytokine-released syndrome (in the presence of alemtuzumab, drug-dependent antibody binds to specific epitopes on platelet surface glycoproteins) or a complement-mediated lysis of circulating platelets (89). While neutropenia in addition, the drug is able to deplete lymphocytes, natural killer cells and monocytes by complement-mediated lysis of leukocytes expressing CD52 glycoprotein on their cell surfaces, leading to leukopenia and/or neutropenia (90, 91).

With regard to natalizumab, the majority of cases concerning adult patients were related to cases of anemia and thrombocytopenia. Data from a longitudinal study reported that the chronic treatment (18 months) was associated with significant modifications in complete blood cell count (increase in mean total white blood cell, lymphocyte, and eosinophil counts as a result of the inhibition in the transmigration of these cells into the central nervous systems that leads to their accumulation in peripheral blood) (92). Natalizumab blocks the alpha-4 subunit of the integrin molecules on leukocytes, leading to their extravasation into the CNS and intestinal tract (93). Contrary to what is observed with other DMTs, natalizumab is associated with an increase in CD4+, CD8+ T cells, CD19+ B cells, and NK cells in serum (94, 95). During natalizumab treatment, an increased release of CD34+ promotor cells from the bone marrow with a consequent increase of absolute lymphocyte counts in serum is observed. This increase tends to stabilize 3–6 months after starting treatment and lasts up to 6 months after discontinuation (96). On the other hand, the pathogenesis leading to thrombocytopenia is unclear, but overall authors of articles describing these cases mainly suggested a drug-induced immune-mediated mechanism, albeit in absence of antibodies against platelet. These effects, together with the fact that natalizumab induces a weakening of the immune systems and that it passes readily through the placenta during the third trimester of pregnancy, may explain the risk of hematological disorders such as anemia and thrombocytopenia in the newborns of mothers exposed to natalizumab. In addition, considering that the newborn’s immune system is under-developed at time of birth, the exposure to natalizumab may further impair its function and render the baby even more susceptible to infections (97, 98). However, notwithstanding a risk of hematological abnormalities in the newborn and spontaneous abortion at the same rate as that of the general population, the use of natalizumab is considered to be safe in pregnancy. The strict monitoring of patients is advisable to minimize the risk of such adverse outcomes.

Data from pivotal trials on ocrelizumab reported a decrease in neutrophil counts in 13–15% of patients with grade 4 neutropenia observed in up to 1% (99). Through the analysis of data reported in the FDA Adverse Event Reporting System (FAERS), Hammer H et al. aimed to identify risk factors of neutropenia in patients treated with ocrelizumab. They identified male sex, younger age and lower bodyweight as factors associated with ocrelizumab-related neutropenia (100). Studies involving rituximab and evaluating bone marrow functionality found that late-onset neutropenia was induced by the white cell line maturation arrest, which in turn is the result of excessive levels of B-cell activating factor (101, 102). As reported by Baird-Gunning (28), late-onset neutropenia can occur also several months after ocrelizumab infusion and it might be caused by alterations in growth factors that drive B-cell production inducing a significant reduction in neutrophils. Since this event is unpredictable and consequently not preventable, blood routine tests represent the main tool for risk mitigation.

Preclinical data on mechanisms underlying fingolimod-induced hematological abnormalities reported that fingolimod transiently increased platelets via S1pr1 activation on megakaryocytes (103), while further data reported that the drug is associated with lymphopenia due to an action on S1P receptor 1 by blocking the egress of lymphocytes from secondary lymphoid organs and preventing them from reaching inflamed tissues (104).

Lastly, few cases concerned the occurrence of hematological disorders in patients treated with IFNs. These DMTs are associated with a 20–30% drop of absolute lymphocyte count due to multiple mechanisms that include a reduction of dendritic cells and a down-regulation of the antigen presentation by antigen-presenting cells (APCs), a reduction in Th17 cells that in turn leads to a reduction of IL-17 release and induction of apoptosis of autoreactive T cells, a reduction of leukocyte migration via the blood–brain barrier into the CNS (105, 106).

Overall, the majority of cases referred to late-onset toxicities, that occurred more than 1 month after the beginning of the therapy with a DMT. Indeed, only 11 studies described the occurrence of early-onset hematological disorders, of which half of them were related to alemtuzumab. Generally, myeloid cytopenia, which include neutropenia, thrombocytopenia, and anemia, are the most common manifestations of drug-related myelotoxicity and the most common reasons for dose modifications or even therapy discontinuation (107). Late-onset neutropenia is confirmed when absolute neutrophil count (ANC) is <1.5 × 10^9^/L and when it develops more than 4 weeks after last drug administration (108). Rarely neutropenia is severe and it can result in neutropenic fever and infection that require patient’s hospitalization, the need for broad-spectrum antibiotics, and the potential sequelae of bacteremia, up to be fatal (109).

AIHA is an acquired autoimmune disorder that develops when autoantibodies develop against self-antigens on the red blood cells, which leads to their destruction. The diagnosis is based on the presence of certain symptoms and laboratory findings including anemia, jaundice, splenomegaly, reticulocytosis, raised serum bilirubin, and a positive direct antiglobulin test (DAT), which detects the presence of antibodies or complement on the red blood cell (RBC) surface (110).

On the other hand, thrombocytopenia is defined in presence of platelet counts <100 × 10^9^/L or > 50% drop in the platelet count from baseline. In severe cases, when the platelet count is <50 × 10^9^/L, there is an increased risk of bleeding that can result in patient’s death. The mechanism underlying the occurrence of drug-induced thrombocytopenia can be either a decrease in platelet production (bone marrow toxicity) or an increased destruction (immune-mediated thrombocytopenia) (111). Many drugs seem to be related to the occurrence of this event, including carbamazepine, ceftriaxone, mirtazapine, oxaliplatin, penicillin, quinine, quinidine, rifampicin, NSAIDs, vancomycin and diuretics (112). Many case reports not included in this review described the occurrence of thrombotic microangiopathy during the treatment with IFN (113–116). Although these cases reported the occurrence of thrombocytopenia, they were not considered for inclusion in this systematic review considering that the reduction in platelets’ count is not the result of a myeloid toxicity rather than other conditions that are not related to suppression of myeloid function.

In conclusion, based on data summarized in this systematic review, the majority of DMTs currently used to treat MS seems to be associated with the occurrence of hematological disorders, even though to a different extent depending on the DMT. For this reason, blood tests need to be carried out before starting the treatment with a specific DMT; for example, due to the risk of ITP, complete blood counts with differential should be obtained prior to initiation of treatment with alemtuzumab and at monthly intervals thereafter until at least 48 months after the last infusion (117). Similarly, to due the risk of lymphopenia, before starting the therapy with dimethyl fumarate, a complete blood count, including lymphocytes, must be performed and repeated every 3 months. The treatment should not be initiated in patients with lymphocyte counts <0.5 × 10^9^ /L. (118) These recommendations are also reported for fingolimod (119), natalizumab, when switching patients from another DMT (120) and ocrelizumab (due to the risk of occurrence of late neutropenia, measurement of blood neutrophils is recommended in patients with signs and symptoms of infection) (121). In general, apart from the regular monitoring of blood cells counts, in order to prevent potentially severe consequences of hematological disorders (infections and bleedings that might lead to patients’ death) and to improve their management, patients receiving DMTs should be educated about the signs and symptoms of myelotoxicity, such as unexplained fatigue, recurrent infections or bleeding (122–127). Indeed, although DMTs-induced hematological disorders seem to be, in the vast majority of cases, self-limiting and rarely associated with serious complications, their early recognition and management is essential for patients’ safety.

## 5 Limitations

This article has some limitations. First of all, the number of studies and included patients was modest, especially for some DMTs such as fingolimod, dimethyl fumarate, cladribine, siponimod and glatiramer acetate. Second, included cases were heterogeneous in terms of hematological disorders, which render the comparison across cases quite difficult. Third, incomplete data reporting was frequent across studies, including information on demographic characteristics (ethnicity, geographic region and education were lacking in the majority of studies), patients’ medical history (timelines of treatments was not frequently reported), and complete/consecutive inclusion of patients for case series. Lastly, apart from cases reporting a positive dechallenge (signs of myelotoxicity disappear after stopping the drug) and rechallenge (signs of myelotoxicity re-appear after introducing for the second time the drug) and for those cases without other clearly stated medical causes, it is not simple to establish a causal relationship for all cases included in this review. Based on these limitations, our results should be interpreted with caution and further *ad hoc* studies are strongly needed to better evaluate the myeloid toxicities of DMTs.

## Data availability statement

The original contributions presented in the study are included in the article, further inquiries can be directed to the corresponding author/s.

## Author contributions

CS: Conceptualization, Data curation, Investigation, Methodology, Writing – original draft, Writing – review & editing. VL: Investigation, Methodology, Writing – review & editing. OA: Data curation, Investigation, Writing – review & editing. DDGC: Investigation, Software, Writing – review & editing. MGS: Investigation, Writing – review & editing. VA: Conceptualization, Investigation, Writing – review & editing. LS: Data curation, Investigation, Writing – review & editing. GTM: Conceptualization, Data curation, Investigation, Writing – review & editing. AC: Conceptualization, Data curation, Writing – original draft, Writing – review & editing.
