# Supplementary material for: Disease-modifying therapies and hematological disorders: a systematic review of case reports and case series
Source: Front Neurol. 2024 Jun 18;15:1386527. doi: 10.3389/fneur.2024.1386527 (PMC11217193; doi:10.3389/fneur.2024.1386527)
Supplement: Supplementary file 1 [file Data_Sheet_1.docx]

**Supplementary Table 1**. Overview of Disease Modifying Therapies (DMTs) used to treat Multiple Sclerosis (MS), their mechanism of action and data from clinical studies reporting the association with myelotoxicities

| **DMT** | **Route/ frequency of administration** | **Mechanism of action of the drug** | **Data on myelotoxicities** |
| --- | --- | --- | --- |
| Alemtuzumab | Intravenous infusion/ 12 mg/day on 5 consecutive days (60 mg total dose) and 12 mg/day on 3 consecutive days administered 12 months after the first treatment course (**1**) | Anti-CD52 monoclonal antibody, targeting T and B cells, monocytes, dendritic cells, and thymocytes | Grades 3 and 4 lymphopenias occurred in 99.9% of patients (**1**), mild neutropenia in 16% of patients, severe neutropenia in 0.6% (**2**) |
| Cladribine | Oral/3.5 mg/kg over 2 years, administered as 1 treatment course of 1.75 mg/kg per year. Each treatment course consists of 2 treatment weeks, one at the beginning of the first month and one at the beginning of the second month of the respective treatment year (**3**) | Anti-pyrimidine and anti-metabolite agent that is biologically active in selected cell types. The drug is able to sustain and target the reduction of circulating B and T lymphocytes that in MS activate a cascade of inflammatory cytokines and antibodies directed against various CNS component | 4 months after treatment initiation in year one, total lymphocyte counts decreased by 42%, while 2 months after the second cycle in year 2, lymphocytes decreased by 58%. Grade 3 lymphopenia occurred in 26% of patients and grade 4 in 0.7% (**4**) |
| Dimethyl fumarate | Oral/ 120 mg/ twice a day. After 7 days, the dose should be increased to the recommended maintenance dose of 240 mg twice a day ([**5**](https://www.ema.europa.eu/en/documents/product-information/tecfidera-epar-product-information_en.pdf)) | It is believed that its mechanism of action involves both nuclear factor erythroid-derived 2-related factor–dependent and independent pathways, which leads to an anti-inflammatory immune response due to type II myeloid cell and Th2 cell differentiation and neuroprotection (**6**) | In the placebo-controlled studies, mean lymphocyte counts decreased by 30% of baseline value over the first year. Lymphocyte counts < 0.5 × 10^9^/L were observed in 6% of patients treated with glatiramer acetate. In clinical studies, 41% of patients treated with the drug had lymphopenia (mild lymphopenia in 28% of patients, moderate lymphopenia in 11% of patients and severe lymphopenia in 2% of patients) (**5**) |
| Glatiramer acetate | Subcutaneous injection /20 mg/day | The drug competes with the MS autoantigen myelin basic protein antigens for binding to class II MHC II, leading to a reduction in helper T cells activation and to a inhibition of the interaction of MHC II with CD4+ molecules located on the surface of helper T cells. This results in a reduced production of pro-inflammatory cytokines (**6**) | Data from a safety analysis covering of all patients with MS who have ever been exposed to the drug in 20 clinical studies reported that shifts in laboratory values were infrequent. Overall, a reduction in laboratory values in >1% of patients were seen in hemoglobin (1.1%) and glucose (1.9%) and an increase in total bilirubin (1.6%) and potassium (1.5%) (**7**). |
| IFN beta-1b | Subcutaneous injection/250 mcg/ Every Other Day | Beta interferons are known to exert autocrine and paracrine actions via activation of the interferon receptor on leukocytes. They induced the production of anti-inflammatory cytokines (**6**) | Hematological abnormalities (leucopenia, lymphopenia, granulocytopenia and thrombocytopenia) are common during the first 6 months of therapy, dose-related, mainly mild and transient (**8**) |
| IFN beta-1a | Intramuscular injection/ 30 mcg/ once a week |  |  |
| S1PR modulators (fingolimod and siponimod) | Fingolimod: Oral/ 0.5 mg/day (**9**)  Siponimod: Oral/0.25 mg/day for two days. The dose is then progressively increased to reach the ‘maintenance’ dose on the sixth day (**10**) | Fingolimod activates lymphocyte S1P1 via high-affinity receptor binding, yet subsequently induces S1P1 down-regulation preventing lymphocyte egress from lymphoid tissues and reducing auto-aggressive lymphocyte infiltration into the CNS.  Siponimod is a second-generation, more selective S1PR1 and S1PR5 modulator and synthetic derivative of fingolimod | In patients receiving fingolimod a dose-dependent decrease in total peripheral lymphocytes to 20–30% of baseline can be observed (**9**).  Siponimod leads to a dose-dependent reduction of peripheral lymphocytes by 70–80%, with a recovery to the normal range within 10 days-4 weeks after treatment discontinuation. Grade 4 lymphopenia was observed in 1% of patients (**10,11**). |
| Natalizumab | Intravenous infusion / 300 mg/once every 4 weeks (**12**) | Anti-α4 integrin monoclonal antibody that prevents lymphocyte migration through the BBB into the CNS | There is a significant increase in various immune cell types, including CD4+ and CD8+ T cells, CD19+ B cells, and NK cells in peripheral blood (**13,14**). After drug discontinuation, circulating lymphocyte levels return to their baseline levels, usually within 16 weeks, while the CD4+/CD8+ ratio in CSF returns to normal within approximately 6 months (**15,16**) |
| Ocrelizumab | Intravenous infusions/ Initial dose of 600 mg is administered as two separate intravenous infusions; first as a 300 mg infusion, followed 2 weeks later by a second 300 mg infusion. Subsequent doses of single 600 mg intravenous infusion every 6 months (**17**) | A humanized anti-CD20 monoclonal antibody with a proposed higher capacity for direct ADCC | After the initial infusion, B cells were significantly reduced within 2 weeks and remained depleted during treatment. About 20.7% of RRMS and 26.3% of PPMS patients experienced a decrease in total lymphocyte counts, mostly with mild lymphopenia. 13% of PMS patients showed a slight decrease in neutrophils while 1% had severe neutropenia (**18**) |
| Teriflunomide | Oral/14 mg/day (**19**) | Teriflunomide targets and inhibits dihydroorotate dehydrogenase, leading to a reduction in the proliferation and function of these specific cells, such as T and B cells (**20**) | Within the initial 6 weeks of treatment, a reduction of 15% in WBC count from the baseline level can be observed. Data from a pooled analysis of clinical trials revealed that mild lymphopenia was not common and no reports of severe lymphopenia have been reported. Grade 1 lymphopenia recovered after approximately 10.6 weeks, while grade 2 after approximately 16.6 weeks (**21**) |

ADCC: antibody-dependent cellular cytotoxicity; BBB: Blood Brain Barrier; CNS: Central Nervous System; CSF: cerebrospinal fluid; MHC: major histocompatibility complex; MS: Multiple Sclerosis; RRMS: Relapsing Remitting MS; PPMS: Primary Progressive MS; WBC: white blood cell

**Supplementary Table 2**. JBI checklist evaluation for case reports included in the systematic review (**22**)

| **First author (ref)** | **Q1** | **Q2** | **Q3** | **Q4** | **Q5** | **Q6** | **Q7** | **Q8** |
| --- | --- | --- | --- | --- | --- | --- | --- | --- |
| Alba Suárez EM (18) | N | N | Y | Y | Y | Y | Y | Y |
| Cisarovsky C (19) | N | Y | Y | Y | Y | Y | Y | Y |
| Najdaghi (20) | N | Y | Y | Y | Y | Y | Y | Y |
| Chan (22) | N | N | Y | Y | Y | Y | Y | Y |
| Koseahmet (23) | N | Y | Y | Y | N | Y | Y | Y |
| Rauniyar (24) | N | U | Y | Y | Y | Y | Y | Y |
| Aitken (25) | N | U | Y | Y | Y | Y | U | Y |
| Sparaco (26) | N | Y | Y | Y | Y | Y | Y | Y |
| Maniscalco (27) | N | N | Y | Y | Y | Y | Y | Y |
| Ganju (28) | N | U | Y | Y | Y | Y | Y | Y |
| Baird-Gunning (29) | N | Y | Y | Y | Y | Y | Y | Y |
| Caldito (31) | Y | U | Y | Y | Y | Y | Y | Y |
| Erlich-Malona (32) | N | Y | Y | Y | Y | Y | Y | Y |
| Auer (33) | N | Y | Y | Y | Y | Y | Y | Y |
| Alnahdi (34) | Y | Y | Y | Y | Y | Y | Y | Y |
| Maniscalco (35) | N | U | Y | Y | Y | Y | Y | Y |
| Tzartos (36) | N | N | Y | Y | Y | Y | Y | Y |
| Zanetta (37) | N | Y | Y | Y | Y | Y | Y | Y |
| Rini (38) | Y | Y | Y | Y | Y | Y | Y | Y |
| Quintanilla-Bordás (39) | N | N | Y | Y | Y | Y | Y | Y |
| Cohen (40) | N | Y | Y | Y | Y | Y | Y | Y |
| Maniscalco (41) | N | Y | Y | Y | Y | Y | Y | Y |
| Vakrakou (42) | N | N | Y | Y | Y | Y | Y | Y |
| Galgani (43) | Y | Y | Y | Y | Y | Y | Y | Y |
| Yiannopoulou (44) | Y | N | Y | Y | Y | Y | Y | Y |
| Di Ioia (45) | N | Y | Y | Y | Y | Y | Y | Y |
| Meunier (46) | N | Y | Y | Y | Y | Y | Y | Y |
| Zecca (47) | N | Y | Y | Y | Y | Y | Y | Y |
| De Masi (52) | N | Y | Y | Y | Y | Y | Y | Y |
| Simone (54) | N | Y | Y | Y | Y | Y | Y | Y |
| Cachia (55) | N | N | Y | Y | Y | Y | Y | Y |
| La Mantia (50) | Y | N | Y | Y | Y | Y | Y | Y |
| Lysandropoulos (58) | N | Y | Y | Y | Y | Y | Y | Y |
| Münzel (59) | Y | Y | Y | Y | Y | Y | Y | Y |
| Saeedi (62) | N | Y | Y | Y | Y | Y | Y | Y |
| Aslam (64) | N | N | Y | Y | Y | Y | Y | Y |
| Bourdin (65) | N | Y | Y | Y | Y | Y | Y | Y |
| Azimi (67) | N | Y | Y | Y | Y | Y | Y | Y |
| Mahmoudi (68) | Y | Y | Y | Y | Y | Y | Y | Y |
| Kim (70) | N | Y | Y | Y | Y | Y | Y | Y |
| Lim (71) | Y | Y | Y | Y | Y | Y | Y | Y |
| Kermode (72) | N | N | Y | Y | Y | Y | Y | Y |
| Marrodan (73) | N | Y | Y | Y | Y | Y | Y | Y |
| Mansoor (74) | N | Y | Y | Y | Y | Y | Y | Y |
| Love (75) | N | N | Y | N | N | N | Y | Y |
| Mukharesh (76) | Y | Y | Y | Y | Y | Y | Y | Y |
| Shaygannejad (77) | N | Y | Y | Y | Y | Y | Y | Y |
| Etemadifar (78) | N | N | Y | N | Y | Y | Y | Y |
| Sagy (80) | N | Y | Y | Y | Y | Y | Y | Y |
| Griggs (81) | N | Y | Y | Y | Y | Y | Y | Y |
| Stosic (82) | Y | Y | Y | Y | Y | Y | Y | Y |
| Sahraian (83) | N | N | N | N | Y | N | N | Y |
| Alanoglu (84) | N | Y | Y | Y | Y | Y | Y | Y |

U: Unclear; Y: Yes; N: No, NA: Not Applicable

**Supplementary Table 3**. JBI checklist evaluation for case series included in the systematic review (**22,23**)

| **First author (ref)** | **N. cases** | **Q1** | **Q2** | **Q3** | **Q4** | **Q5** | **Q6** | **Q7** | **Q8** | **Q9** | **Q10** |
| --- | --- | --- | --- | --- | --- | --- | --- | --- | --- | --- | --- |
| Rossi (21) | 3 | Y | Y | Y | U | U | N | Y | Y | U | NA |
| Yuen (48) | 3 | U | Y | Y | U | U | N | Y | Y | U | NA |
| Gaitán (49) | 2 | U | Y | Y | N | N | N | Y | Y | N | NA |
| Monteleone (53) | 2 | U | Y | Y | N | N | N | Y | Y | N | NA |
| Tanaka (60) | 3 | N | Y | Y | N | N | N | N | Y | N | NA |
| Midaglia (61) | 2 | U | Y | Y | N | N | N | Y | Y | N | NA |
| Nabavi (63) | 2 | U | Y | Y | N | N | N | Y | Y | N | NA |
| Pang (66) | 9 | N | Y | Y | N | N | N | N | Y | N | NA |
| Beigneux (69) | 3 | U | Y | Y | N | N | N | Y | Y | N | NA |
| Obermann (79) | 2 | U | Y | Y | N | N | N | Y | Y | N | NA |

U: Unclear; Y: Yes; N: No, NA: Not Applicable

**References**

1. European Medicines Agency. Lemtrada® Summary of Product Characteristics. Available at: https://www.ema.europa.eu/en/documents/product-information/lemtrada-epar-product-information_en.pdf (accessed on 25-10-2023)
2. Vakrakou AG, Tzanetakos D, Valsami S, Grigoriou E, Psarra K, Tzartos J, Anagnostouli M, Andreadou E, Evangelopoulos ME, Koutsis G, Chrysovitsanou C, Gialafos E, Dimitrakopoulos A, Stefanis L, Kilidireas C. A case of Alemtuzumab-induced neutropenia in multiple sclerosis in association with the expansion of large granular lymphocytes. BMC Neurol. 2018;18(1):178.
3. European Medicines Agency. Mavenclad® Summary of Product Characteristics. Available at: <https://www.ema.europa.eu/en/documents/product-information/mavenclad-epar-product-information_en.pdf> (accessed on 25-10-2023)
4. European Medicines Agency. Mavenclad—assessment report. Available at: [https://www.ema.europa.eu/en/documents/assessment-report/mavenclad-epar-public-assessment-report_en.pdf. Accessed 29 Nov 2019](https://www.ema.europa.eu/en/documents/assessment-report/mavenclad-epar-public-assessment-report_en.pdf.%20Accessed%2029%20Nov%202019) (accessed on 25-10-2023)
5. European Medicines Agency. Tecfidera® Summary of Product Characteristics. Available at: <https://www.ema.europa.eu/en/documents/product-information/tecfidera-epar-product-information_en.pdf> (accessed on 25-10-2023)
6. Pardo G, Jones DE. The sequence of disease-modifying therapies in relapsing multiple sclerosis: safety and immunologic considerations. J Neurol. 2017 Dec;264(12):2351-2374. doi: 10.1007/s00415-017-8594-9. Epub 2017 Sep 6. Erratum in: J Neurol. 2017 Dec;264(12 ):2375-2377. PMID: 28879412; PMCID: PMC5688209.
7. Ziemssen T, Ashtamker N, Rubinchick S, Knappertz V, Comi G. Long-term safety and tolerability of glatiramer acetate 20 mg/ml in the treatment of relapsing forms of multiple sclerosis. Expert Opin Drug Saf. 2017 Feb;16(2):247-255. doi: 10.1080/14740338.2017.1274728. PMID: 27989217.
8. Rieckmann P, O'Connor P, Francis GS, Wetherill G, Alteri E. Haematological effects of interferon-beta-1a (Rebif) therapy in multiple sclerosis. Drug Saf. 2004;27(10):745-56. doi: 10.2165/00002018-200427100-00005. PMID: 15350158.
9. European Medicines Agency. Gilenya® Summary of Product Characteristics. Available at: https://www.ema.europa.eu/en/documents/product-information/gilenya-epar-product-information_en.pdf (accessed on 25-10-2023)
10. https://www.ema.europa.eu/en/documents/overview/mayzent-epar-medicine-overview_en.pdf
11. Kappos L, Bar-Or A, Cree BAC, Fox RJ, Giovannoni G, Gold R, Vermersch P, Arnold DL, Arnould S, Scherz T, Wolf C, Wallstrom E, Dahlke F, Investigators EC. Siponimod versus placebo in secondary progressive multiple sclerosis (EXPAND): a double-blind, randomised, phase 3 study. *Lancet.*2018;391(10127):1263–1273.
12. European Medicines Agency. Tysabri® Summary of Product Characteristics. Available at: https://www.ema.europa.eu/en/documents/product-information/tysabri-epar-product-information_en.pdf (accessed on 25-10-2023)
13. Saure C, Warnke C, Zohren F, Schroeder T, Bruns I, Cadeddu RP, Weigelt C, Fischer U, Kobbe G, Hartung HP, Adams O, Kieseier BC, Haas R. Natalizumab and impedance of the homing of CD34+ hematopoietic progenitors. Arch Neurol. 2011;68(11):1428–1431.
14. Warnke C, Smolianov V, Dehmel T, Andree M, Hengel H, Zohren F, Arendt G, Wiendl H, Haas R, Hartung HP, Adams O, Kieseier BC. CD34+ progenitor cells mobilized by natalizumab are not a relevant reservoir for JC virus. Mult Scler. 2011;17(2):151–156.
15. Koudriavtseva T, Sbardella E, Trento E, Bordignon V, D’Agosto G, Cordiali-Fei P. Long-term follow-up of peripheral lymphocyte subsets in a cohort of multiple sclerosis patients treated with natalizumab. Clin Exp Immunol. 2014;176(3):320–326.
16. Stuve O, Marra CM, Bar-Or A, Niino M, Cravens PD, Cepok S, Frohman EM, Phillips JT, Arendt G, Jerome KR, Cook L, Grand’Maison F, Hemmer B, Monson NL, Racke MK. Altered CD4+/CD8+ T-cell ratios in cerebrospinal fluid of natalizumab-treated patients with multiple sclerosis. *Arch Neurol.*2006;63(10):1383–1387.
17. European Medicines Agency. Ocrevus® Summary of Product Characteristics. Available at: <https://www.ema.europa.eu/en/documents/product-information/ocrevus-epar-product-information_en.pdf> (accessed on 25-10-2023)
18. EMA. Ocrevus—EPAR—product information. [https://www.ema.europa.eu/en/documents/product-information/ocrevus-epar-product-information_en.pdf. (accessed on 25-10-2023)](https://www.ema.europa.eu/en/documents/product-information/ocrevus-epar-product-information_en.pdf.%20Accessed%209%20Dec%202019)
19. European Medicines Agency. Aubagio® Summary of Product Characteristics. Available at: https://www.ema.europa.eu/en/documents/product-information/aubagio-epar-product-information_en.pdf (accessed on 25-10-2023)
20. Bar-Or A, Pachner A, Menguy-Vacheron F, Kaplan J, Wiendl H. Teriflunomide and its mechanism of action in multiple sclerosis. Drugs. 2014;74(6):659–674
21. Comi G, Miller AE, Benamor M, Truffinet P, Poole EM, Freedman MS. Characterizing lymphocyte counts and infection rates with long-term teriflunomide treatment: pooled analysis of clinical trials. Mult Scler. 2019;7:1352458519851981.
22. JBI Critical Appraisal Tool. Available at: https://jbi.global/critical-appraisal-tools (accessed on 4-12-2023)
23. Munn Z, Barker TH, Moola S, Tufanaru C, Stern C, McArthur A, Stephenson M, Aromataris E. Methodological quality of case series studies: an introduction to the JBI critical appraisal tool. JBI Evidence Synthesis. 2020;18(10):2127-2133
